# Characterization and comparative analyses of transcriptomes of cloned and *in vivo* fertilized porcine pre-implantation embryos

**DOI:** 10.1242/bio.039917

**Published:** 2019-04-05

**Authors:** Xiaoyan He, Cheng Tan, Zicong Li, Chengfa Zhao, Junsong Shi, Rong Zhou, Xingwang Wang, Gelong Jiang, Gengyuan Cai, Dewu Liu, Zhenfang Wu

**Affiliations:** 1 National Engineering Research Center for Breeding Swine Industry, College of Animal Science, South China Agricultural University, Guangzhou 510642, China; 2 Wen's Group Academy, Wen's Foodstuff Group Co., Ltd, Yunfu 527400, China, China

**Keywords:** Pig, Pre-implantation embryos, RNA-seq, Somatic cell nuclear transfer

## Abstract

Somatic cell nuclear transfer (SCNT) is the only method known to rapidly reprogram differentiated cells into totipotent embryos. Most cloned embryos become arrested before implantation and the details of the underlying molecular mechanism remain largely unknown. Dynamic regulation of the transcriptome is a key molecular mechanism driving early embryonic development. Here, we report comprehensive transcriptomic analysis of cloned embryos (from Laiwu and Duroc pigs) and *in vivo* fertilized embryos (from Duroc pigs) using RNA-sequencing. Comparisons between gene expression patterns were performed according to differentially expressed genes, specific-expressed genes, first-expressed genes, pluripotency genes and pathway enrichment analysis. In addition, we closely analyzed the improperly expressed histone lysine methyltransferases and histone lysine demethylases during cell reprogramming in cloned embryos. In summary, we identified altered gene expression profiles in porcine cloned pre-implantation embryos in comparison to normal *in vivo* embryos. Our findings provide a substantial framework for further discovery of the epigenetic reprogramming mechanisms in porcine SCNT embryos.

## Abstract (precis)

**Summary:** Comparative transcriptome analyses of cloned and *in vivo* fertilized pre-implantation embryos: transcriptional defects and reprogramming barriers in porcine somatic cell nuclear reprogramming.

## INTRODUCTION

Somatic cell nuclear transfer (SCNT) is the only available technique that allows the transformation of a single somatic cell into a totipotent embryo that can further develop into an individual animal (Polejaeva et al., 2000; Wilmut et al., 2002, 1997). However, since overall cloning efficiency is still extremely low, this limits the large-scale application of the SCNT technique in basic research, agriculture and human medicine (Loi et al., 2016; Meissner and Jaenisch, 2006; Whitworth and Prather, 2010). Most of the cloned embryos die before they develop into blastocysts. In mice, only about half of SCNT embryos can develop into blastocysts, and in livestock, such as pig, cattle and sheep, blastocyst rates are even lower (<30%) (Khan et al., 2018; Li et al., 2013a; Min et al., 2015; Ogura et al., 2013; Srirattana and St John, 2017).

The domestic pig is not only an important livestock species but is also the newest biomedical animal model owing to its physiological and anatomical similarities with humans. Pigs have even been considered as a primary model for xenotransplantation (Schook et al., 2005; Schuurman and Patience, 2013). To date, only a few studies have utilized genome-wide profiling of gene expression and epigenetic reprogramming dynamics in porcine pre-implantation embryos (Cao et al., 2014; Zhong et al., 2018; Zhou et al., 2014). It is therefore important to conduct a thorough and comprehensive evaluation of transcriptional control in porcine embryos. Additionally, molecular defects associated with assisted reproductive technologies and their impact on porcine pre-implantation, especially in SCNT technology, remain largely unknown (Prather et al., 2014; Zou et al., 2016). Deep transcriptome sequencing can offer a rapid and effective approach for comparing gene expression profiles between cloned and *in vivo* fertilized porcine embryos, and for exploring the abnormal functional and regulatory networks in the cloned porcine embryos. This approach is likely to provide new insight into understanding of the mechanisms of nuclear reprogramming in pigs, and may also suggest critical clues on how to improve pig SCNT efficiency for future studies.

In the present study, next-generation RNA-sequencing (RNA-Seq) was utilized to generate genome-wide transcriptome profiles of cloned and *in vivo* fertilized porcine embryos in seven consecutive points in time. We examined gene expression patterns during the first week of porcine embryo development, including maternal-zygotic transition (MZT) and embryonic genome activation (EGA) processes. We identified differentially expressed genes (DEGs) between the cloned and *in vivo* fertilized embryos from distinct stages of development and uncovered associated cellular signaling pathways and upstream transcription factors (TFs). Finally, our findings and database provide a fundamental resource for better understanding of the reprogramming process as well as the mechanism of developmental defects in the cloned embryos.

## RESULTS

### Experiment overview

**Fig. 1.**
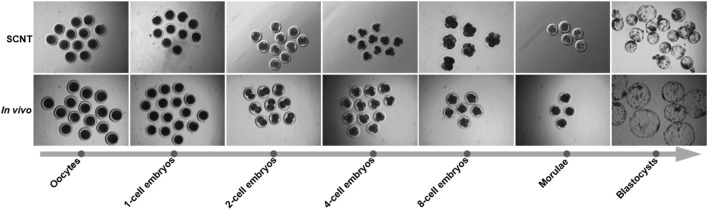
**Microscopy imaging of porcine *in vivo* and *in vitro* matured oocytes and pre-implantation embryos.** From left to right are oocytes, 1-cell embryos, 2-cell embryos, 4-cell embryos, 8-cell embryos, morulae and blastocysts which were derived from artificial insemination or SCNT procedures.

We collected cloned embryos from a cell line of Chinese pig breed of Laiwu (NT-LW group), cloned embryos from a cell line of Duroc pig (NT-D group) and *in vivo* fertilized embryos of Duroc pig (IV-D group) for whole-genome transcriptome sequencing. Embryos used for sequencing included 1-cell embryos, 2-cell embryos, 4-cell embryos, 8-cell embryos, morulae and blastocysts (Fig. 1, Table 1). In addition, we sequenced the Laiwu and Duroc donor cells, *in vivo* oocytes arrested in the metaphase of the second meiotic division (MII) from Duroc sows, and *in vitro* MII oocytes from hybrid sows which were used for the nuclear transfer receptors. A total of 25 samples were sequenced to analyze the dynamic changes in gene expression in the early stages of three different groups of embryos (Table S1). Furthermore, two biological replicates were added for the sequencing of oocytes, 4-cell embryos, 8-cell embryos and blastocysts from the NT-D and IV-D groups and three biological replicates were added for the sequencing of Duroc donor cells. A total of 27 samples were analyzed for more in-depth comparison of the gene expression profiles in the four critical stages of the early embryos (Table S2).

**Table 1.**
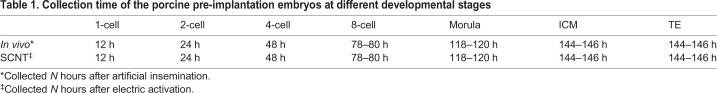
**Collection time of the porcine pre-implantation embryos at different developmental stages**

By using the Illumina HiSeq sequencing platform, we analyzed a total of 44 transcriptome samples, averaging more than 51.2 million reads per sample with a read length of 150 bp (Tables S3 and S4). More than 90% (91.5%–98.0%) of clean reads were mapped to the pig reference genome assembly (Tables S5 and S6) and approximately 11.4×10^3^ genes (8.5×10^3^–14.2×10^3^) out of 37.4×10^3^ RefSeq genes were expressed (Tables S7 and S8). A separate comparative transcriptomic analysis was performed on the 25 and 27 samples mentioned above. These data sets were defined as the 25-sample dataset and 27-sample dataset, and corresponding results were presented separately.

### Dynamic transcriptional profiles during the pre-implantation development of porcine embryos

**Fig. 2.**
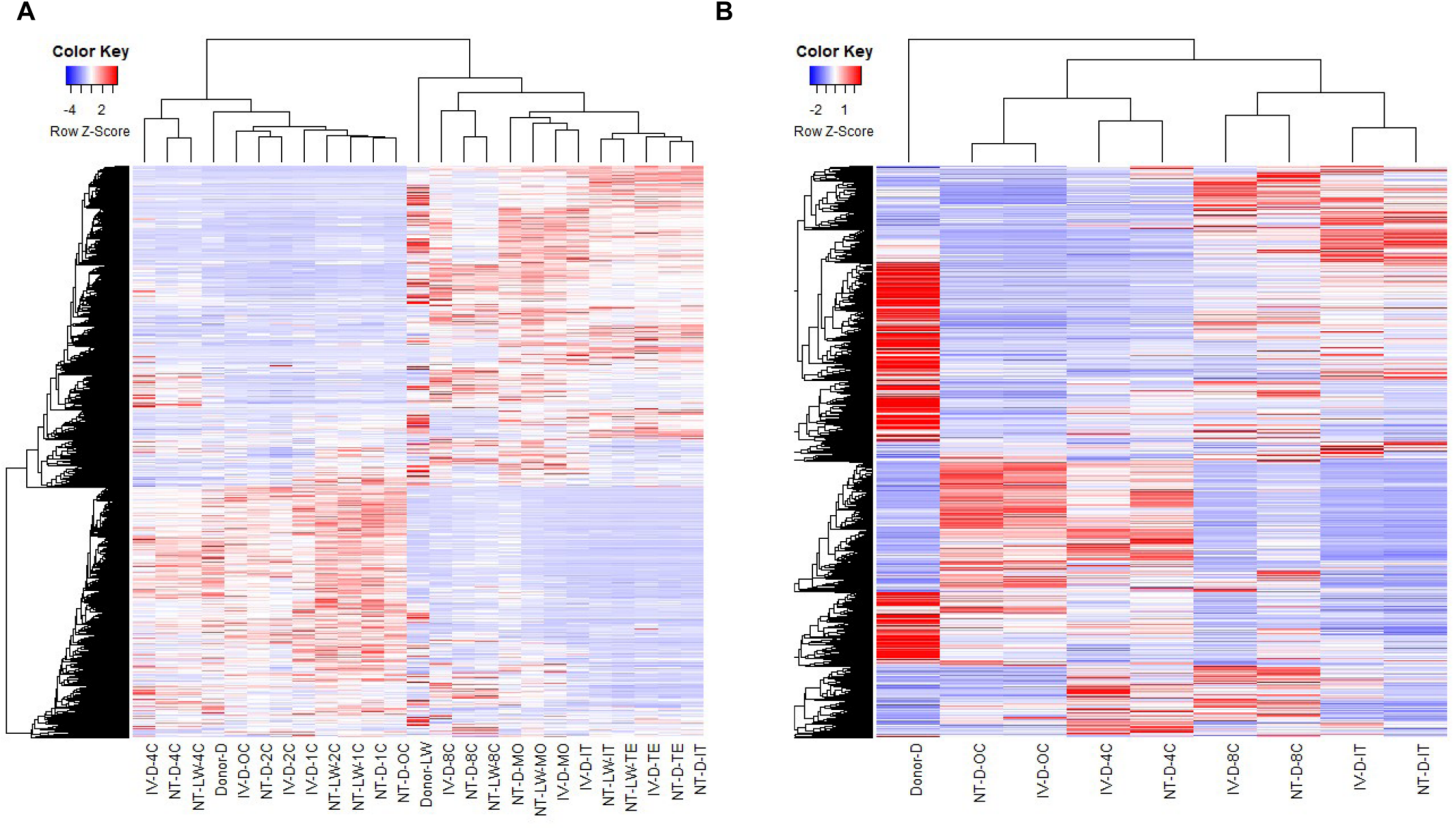
**Unsupervised clustering of the transcriptome of two breeds of cloned and *in vivo* pre-implantation embryos.** (A) Unsupervised hierarchical clustering dendrogram of the transcriptome of all samples without biological replicates in the 25-sample dataset. (B) Hierarchical clustering dendrogram of the transcriptome of all samples with three biological replicates in the 27-sample dataset.

To determine whether gene expression profiles were linked with the developmental stages, we analyzed RNA-Seq data of the oocytes and embryos by unsupervised hierarchical clustering. The results of the 25-sample dataset indicated that the gene expression pattern was similar from the oocytes to 4-cell embryos and that the greatest difference in gene expression was observed between the 4-cell and 8-cell embryos, which can likely be explained by the major MZT process (Fig. 2A). In the 27-sample dataset, cloned and *in vivo* derived embryos that clustered together were at the same developmental stage, and the developmental features of the NT-D and IV-D embryos were consistent with the 25-sample dataset (Fig. 2B). Next, the principal component analysis was conducted on the 27-sample dataset (Fig. S1), revealing that the biological replicates of the 4-cell embryos were relatively variable. Since the 4-cell stage is the main activation period of the zygote genome, gene expression rapidly changes, likely explaining the observed variability. The gene expression level of the biological replicates of oocytes, 8-cell embryos, morulae and blastocysts were closely correlated, which showed correlation coefficients (R^2^) between 0.878 and 0.979, but the correlation was only slightly lower among 4-cell embryos, ranging from 0.787 to 0.889 (Table S9).

### DEGs in two successive developmental stages of porcine early embryos

**Fig. 3.**
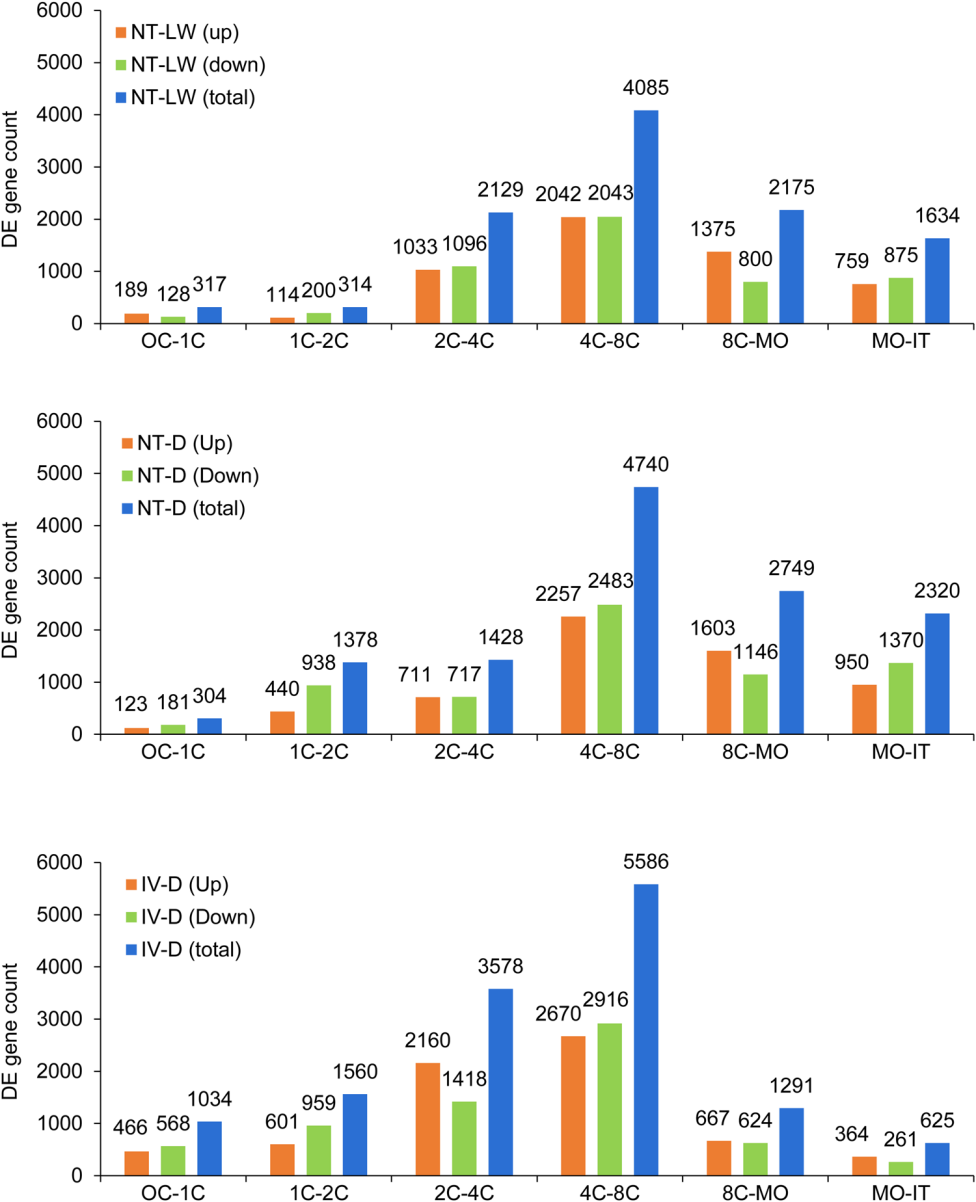
**Numbers of differentially expressed (DE) genes between two successive stages in cloned and *in vivo* embryos.** (A­–C) Histograms of the number of upregulated, downregulated and total DEGs between two successive stages in NT-LW, NT-D and IV-D groups of embryos, respectively. Genes with an adjusted *P*-value<0.005 and the absolute value of log_2_ (fold-change)>1 were found to be differentially expressed between two successive stages.

Mammalian embryo pre-implantation development undergoes a process from maternal to zygotic control which requires maternal transcript degradation and zygotic transcript activation. In order to compare the transcriptional profiles among developmental stages, the upregulated and downregulated DEGs in the successive developmental stages of the NT-LW, NT-D and IV-D embryo groups at seven cleavage stages were calculated (Fig. 3A–C). In IV-D embryos, the number of DEGs in the 4-cell to 8-cell stage was the highest with 2670 upregulated genes and 2916 downregulated genes. Next, there were 2160 upregulated and 1418 downregulated genes in the 2-cell to 4-cell embryos. In other stages, the number of upregulated or downregulated genes was less than 1000. Overall, these results suggest that porcine zygotic genome activation process occurs mainly during the 2-cell to 8-cell stage. Compared with the development of cloned embryos, the greatest difference was reflected in the total number of DEGs, which was drastically reduced from the 2-cell to 4-cell stage. There were 711 upregulated and 717 downregulated genes in the 2-cell to 4-cell NT-D embryos, and 1033 upregulated and 1096 downregulated genes in the NT-LW embryos. Interestingly, comparing 8-cell to morula and morula to blastocyst stage, the total number of DEGs in the cloned embryos was significantly higher than in IV-D embryos. This result suggests that partial waves of early transcriptional activation appear to be delayed in pig SCNT, and so it may lead to the deaths of many cloned embryos before 8-cell stage.

Ingenuity pathway analysis (IPA, Ingenuity Systems) software was used for the core analysis of the DEGs in two successive early stages of porcine embryo development, and we then performed a comparative analysis of the three groups of embryos. We searched the main canonical pathways that were predicted to be activated or inhibited in each stage of embryonic development. We found that most of the enriched cell signaling pathways were inhibited. However, between the 8-cell stage and blastocyst stage, most of the enriched pathways were activated. Visualization of pathway by activity analysis z-scores while sorted by score, the top five enriched pathways were EIF2 signaling, RhoA signaling, RhoGDI signaling, regulation of actin-based motility by Rho and Integrin signaling (Fig. S2A). At the same time, visualization of pathways was performed using Fisher's exact test *P*-values. Criteria for statistical significance were *P*-value<0.001, 28 terms of significant signaling pathways were enriched (Fig. S2B). Under these conditions, the top five significant enriched pathways were EIF2 signal, protein ubiquitination, oxidative phosphorylation, mitochondrial dysfunction and regulation of eIF4 and p70S6K signaling. Dozens of upstream TFs affecting the sequential development of the successive cleavage stages of porcine early embryos were analyzed (*P*-value<0.001 and |z-score|>2). Among them, the top ten significant TFs derived from IPA analysis were *NFE2L2*, *MYC*, *XBP1*, *MYCN*, *KDM5A*, *PPARGC1A*, *E2F1*, *RB1*, *TP53* and *NUPR1* (Fig. S2C). This result suggests that these genes may play important roles during early porcine embryonic development.

### Stage-specific, first-expressed genes in early development of the cloned and fertilized pre-implantation embryos

**Fig. 4.**
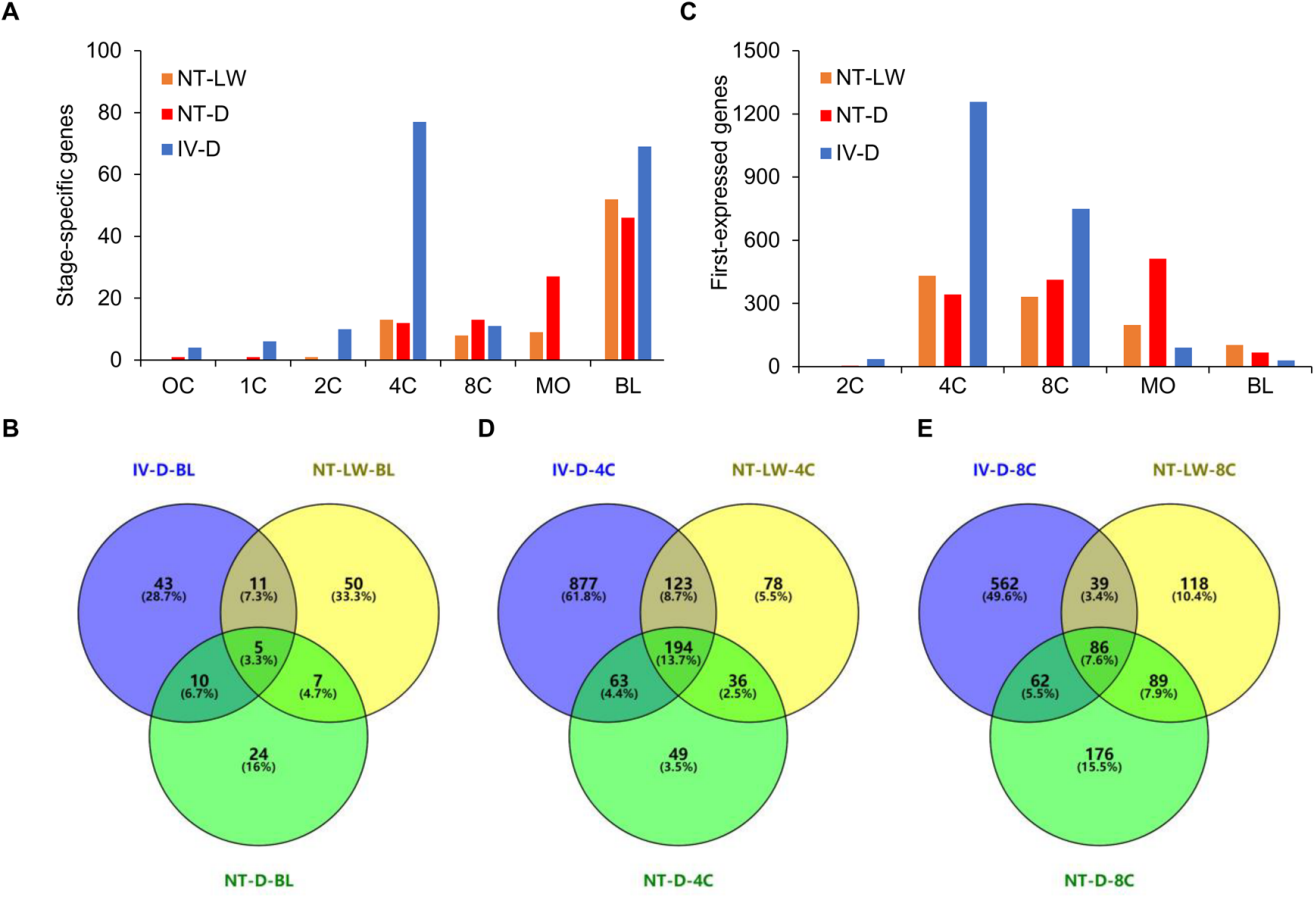
**Identification of stage-specific genes and first-expressed genes.** (A) Histogram of the number of stage-specific genes in NT-LW, NT-D and IV-D embryos. Stage-specific genes are defined as those expressed solely during one stage. (B) Venn diagram for the stage-specific genes in the three groups of blastocysts. (C) Histogram of the number of genes activated at the respective embryonic stages as detected by first appearance of specific transcripts. (D,E) Venn diagrams for the first-expressed genes in the three groups of embryos at the 4-cell and 8-cell stages, respectively.

Further analysis was conducted to identify genes that were expressed uniquely in one of the seven embryonic developmental stages. To identify stage-specific genes is important for understanding the molecular basis of transcriptional control of developmental processes. The stage-specific genes were filtered according to the three restrictive conditions, where the gene fragments per kilobase per million mapped fragments (FPKM) value was >5 at a particular stage, FPKM value was <1 at all other stages and genes had accession numbers in the Sscrofa11.1 reference genome (Fig. 4A; Table S10). The 4-cell and morula stages were the two stages with the greatest numbers of stage-specific genes. Specifically, there were 77 genes uniquely expressed in the IV-D group at the 4-cell stage. Among these, only 12 genes had FPKM value>5 in the NT-LW 4-cell embryos, and only 15 genes had FPKM value>5 in the NT-D 4-cell embryos. Moreover, *in vivo* derived 4-cell embryos specifically expressed two types of U2 and six types of U6 small nuclear RNA (snRNA). As a main component of the RNA splicing during eukaryotic post-transcriptional processing, snRNA is involved in the processing of mRNA precursors and RNA regulation in early embryos. Additionally, there were five genes encoding nuclear TFs, including *OLIG3*, *HMX2*, *VAV1*, *MYT1L* and *FOXG1*, in which the highest level of gene expression was detected from the *OLIG3* gene. *OLIG3* is an important 4-cell stage-specific functional gene, which is a transcriptional co-repressor factor of RNA polymerase II and also the target gene for approximately 90 different miRNAs. Moreover, *OLIG3* also regulates the transcription of DNA promoters and has important biological functions in regulating gene transcription. The FPKM value for this gene in the cloned 4-cell embryos was significantly lower compared to normal embryos, which may lead to the developmental inefficiency of porcine cloned embryos. In blastocysts, the numbers of stage-specific genes were 73, 46 and 69 in the NT-LW, NT-D and IV-D groups, respectively. Among these, only five genes were specifically expressed in all embryo groups (Fig. 4B), suggesting that gene expression patterns at the blastocyst stage are still quite unique between embryo groups.

Next, we identified genes that were activated during early embryonic development. Statistical analysis of the number of first-expressed genes is a good way for understanding the time course of EGA. Genes were considered first-expressed in embryos when the FPKM value was >1 at a particular stage and <1 at all previous cleavage stages, with the adjusted *P*-value<0.05 when compared to the previous time point (Fig. 4C). Application of these filtering criteria in the IV-D group identified 36 genes to be first-expressed at the 2-cell stage, 1257 genes at the 4-cell stage, 749 genes at the 8-cell stage, 91 genes at the morula stage, and 30 genes at the blastocyst stage. The first-expressed genes in each stage of the NT-LW and NT-D embryos were also filtered out (Table S11). We performed a comparative analysis of the first-expressed genes in the three groups at the 4-cell and 8-cell stages (Fig. 4D,E). The results indicated that large-scale activation of genes in the cloned embryos was likely delayed relative to normal embryos. Specifically, up to 512 genes were first-expressed at the morula stage in the NT-D group, indicating serious delay in the genome activation.

### The pattern of pluripotency gene expression during early porcine embryonic development

**Fig. 5.**
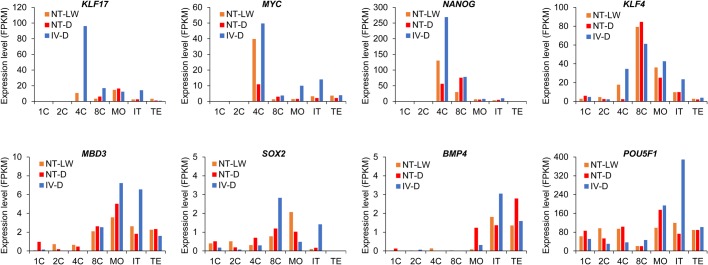
**Expression patterns of eight pluripotency genes.** Each histogram shows the patterns of variability in the FPKM values of a pluripotent gene during early embryonic development. The difference in expression of this gene in NT-LW, NT-D and IV-D embryos can be easily found from the diagrams.

The development of mammalian early embryos critically depends on appropriate expression of pluripotency genes. In order to identify the dynamics of activation of the pluripotency program during porcine embryonic development, the expression patterns of eight pluripotency genes in porcine embryos were analyzed (Fig. 5). Our results showed that the pluripotency networks of early porcine embryos were quite different from mouse and human embryos in earlier reports (Niakan et al., 2012). *KLF17*, *MYC* and *NANOG* were activated and reached the highest level of expression in fertilized 4-cell embryos. Importantly, the expression levels of these three genes were lower in the cloned embryos and particularly the lowest in the Duroc cloned embryos. *KLF4* gene was activated at the 4-cell stage and the expression level was the highest at the 8-cell stage. The expression level of *KLF4* was also significantly reduced in the cloned 4-cell embryos, and was the lowest in the Duroc cloned embryos. The *MBD3* and *SOX2* genes were activated at the 8-cell stages, and were expressed at low levels in the early porcine embryos. The *BMP4* gene was only weakly expressed in blastocysts and not expressed at all during other stages. The expression of *POU5F1* gene was high in embryos from the 1-cell to blastocyst stage and reached the highest level in the fertilized blastocysts and the NT-LW blastocysts. However, in the Duroc cloned embryos, expression of *POU5F1* was the highest at the morula stage. These data suggest that the differences in pluripotent gene expression between the normal embryos and Duroc cloned embryos are relatively greater compared to the Laiwu cloned embryos, and the dysregulation of embryonic cell renewal and differentiation may be more serious in the Duroc cloned embryos.

### Identification of specific transcripts of the inner cell mass in porcine blastocysts

**Fig. 6.**
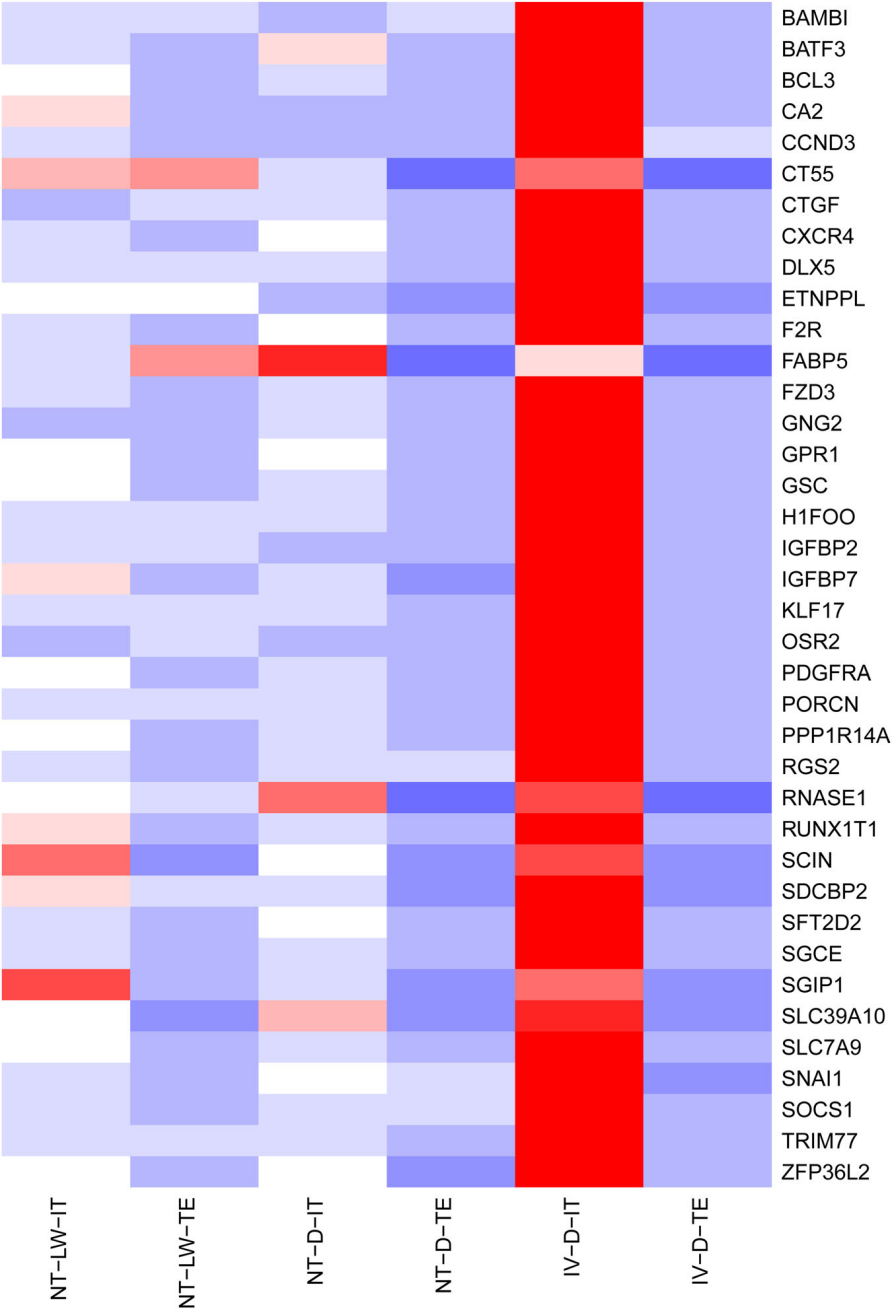
**Heatmap of ICM markers clustering.** Highly expressed genes are shown in red, and minimally expressed genes are shown in blue. Thirty-eight specific transcripts in the ICM (FPKM<1 in TE and FPKM>10 in IT) were identified. The level of expression of most of these genes were significantly reduced in the ICM of NT-LW and NT-D embryos, and they were not expressed in the TE of NT-LW and NT-D embryos.

The earliest differentiation event in mammalian embryos occurs at the blastocyst stage when totipotent blastomeres differentiate into either pluripotent inner cell mass (ICM) or multipotent trophectoderm (TE). The ICM is the source of embryonic stem cells capable of forming all cell types within the embryo, while the TE give rise to the placenta. Cell fate commitment towards ICM or TE is under the control of specific TFs, so we sought to identify the underlying TFs responsible for first lineage segregation. It is technically very challenging to effectively separate the ICM and TE from porcine blastocysts. In this study, porcine blastocysts were cut into two parts under a stereoscopic microscope using a hand-held microdissection knife, where one half contained ICM and TE (IT), and the other only contained TE. Using the transcriptome data of the IV-D embryos, 38 specific transcripts of ICM (FPKM<1 in TE and FPKM>10 in IT) were identified (Fig. 6), and included the following TFs: *GSC*, *RUNX1T1*, *SNAI1*, *BATF3*, *BCL3*, *DLX5*, *OSR2*, *KLF17* and *ZFP36L2*. These exclusively expressed TFs not only serve as markers to distinguish between the ICM and the TE, but also may play crucial roles in first cell fate decision.

### Comparison of gene expression between the cloned and *in vivo* fertilized embryos from the 25-sample dataset

**Fig. 7.**
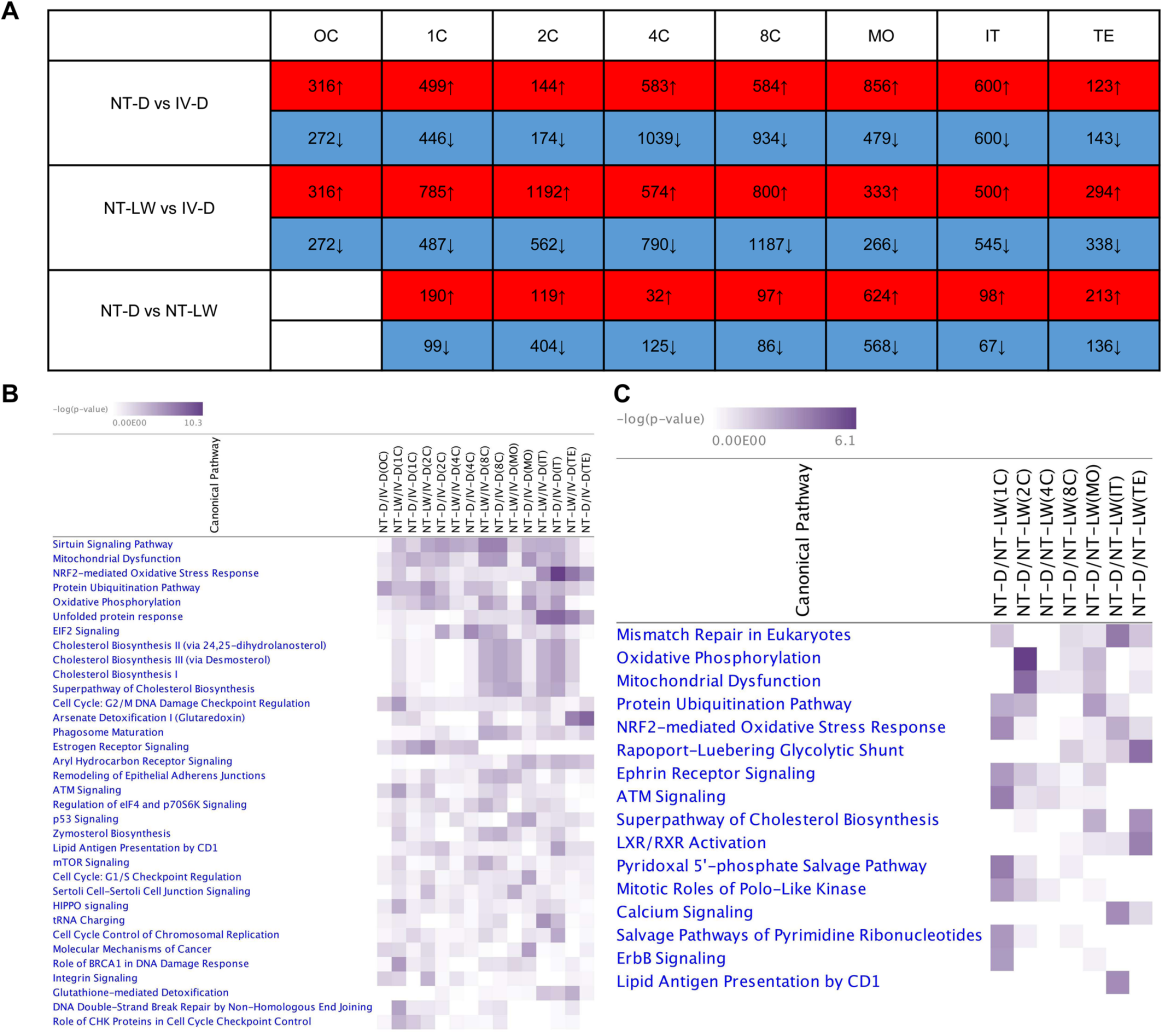
**Comparison of gene expression levels and associated pathways between the cloned and *in vivo* fertilized embryos from the 25-sample dataset.** (A) The number of DEGs between each developmental stage of NT-D, NT-LW and IV-D embryos. (B) Over-representation of IPA canonical pathway annotation terms for DEGs between the cloned and *in vivo* embryos where the criterion for statistical significance was *P*-value<0.0001. (C) Over-representation of IPA canonical pathway annotation terms for DEGs between Laiwu and Duroc cloned embryos at each cleavage stage. Criterion for statistical significance was *P*-value<0.001.

To identify the DEGs between the cloned and *in vivo* fertilized embryos in seven consecutive points in time, we performed pairwise comparison of the transcriptome of NT-D, NT-LW and IV-D embryos from the 25-sample dataset. The number of DEGs between the same cleavage stages of NT-D, NT-LW and IV-D embryos was counted (Fig. 7A). There were many more abnormally expressed genes in the cloned embryos of the two varieties than in normal embryos, but there were far fewer DEGs between the two varieties of cloned embryos. Pathway clustering analysis was performed using an IPA comparative analysis module. Filtering with *P*-value<0.0001, and the top five pathways in which the DEGs of the two groups of cloned embryos and normal embryos were most significantly enriched were sirtuin signaling, mitochondrial dysfunction, NRF2-mediated oxidative stress response, protein ubiquitination, and oxidative phosphorylation pathway (Fig. 7B). The TFs (*P*-value<0.001 and |z-score|>2) enriched from IPA pathways included *XBP1*, *MYC*, *E2F1*, *NFE2L2*, *CDKN2A*, *ATF4*, *TP53*, *E2F*, *HNF4A*, *SREBF1*, *NUPR1*, *TP73*, *TP63*, *TCF3*, *CCND1*, *FOXO3*, *RBL1*, *E2F3*, *CREB1*, *FOXM1*, *SREBF2*. These upstream regulatory factors should be important factors in regulating the cell reprogramming process involved in early embryo development in pigs. The signaling pathways associated with the DEGs in the same cleavage stages of Laiwu and Duroc cloned embryos were also clustered, and the five most profoundly enriched pathways were mismatch repair in eukaryotes, oxidative phosphorylation, mitochondrial dysfunction, protein ubiquitination, and NRF2-mediated oxidative stress response (Fig. 7C). The upstream TFs obtained by IPA analysis (*P*-value<0.05 and |z-score|>2) included *CREB1*, *TP53*, *SMARCA4*, *SREBF1*, *XBP1*, *PDX1*, *NOBOX*, *TCF3*, *CDKN2A*, *CCND1*, *HSF1*, *E2F3*, *SREBF2*, *CEBPA*, *SIM1*, *ARNT2*, *ASXL1*, *BHLHA15*, *TBX2*, *MKL2* and *MYOCD*. These genes may be important factors induced the difference in the transcriptional regulation mechanisms between cloned Laiwu and Duroc embryos.

### Comparison of gene expression between the cloned and *in vivo* fertilized embryos from the 27-sample dataset

Next, we performed differential expression analysis between NT-D and IV-D embryos at four stages from the 27-sample dataset. The RNA-Seq experiment encompassed three biological replicates. At the oocyte stage, there were 39 significant DEGs between the NT-D and IV-D groups, of which 35 were upregulated and four were downregulated (Table S12). Only ten DEGs had gene names in the Sscrofa11.1 reference genome. In IPA network analysis results, these ten genes were clustered into one signal network which was associated with cell growth, proliferation, survival and death, and the core controlling factors in the network were *MYC*, *POU5F1* and *TP53*. Among these ten genes, only *TET1* was downregulated in *in vitro* mature oocytes. Interestingly, 5mC-specific dioxygenase *TET1* protein plays an important role in regulating meiosis in mouse oocytes, and mediates DNA demethylation and subsequent activation of a subset of meiotic genes (Yamaguchi et al., 2012). Importantly, *TET1* deficiency can cause meiotic abnormalities of *in vitro* cultured oocytes, which can likely affect the development of porcine cloned embryos. Additionally, we found the upregulated *DRAP1* gene to belong to the nuclear transcription regulators and regulates DNA promoters, RNA polymerase II and NODAL signaling pathways. The NODAL signaling pathway, regulated by *DRAP1*, *FOXH1* and *OCT4*, plays an important biological role in oocyte maturation and early embryonic development (Cao et al., 2008).

At the 4-cell stage, there was a total of 488 DEGs between the NT-D and IV-D groups, including 57 upregulated and 431 downregulated genes, while only 64 DEGs had gene names in the Sscrofa11.1 reference genome (Table S13). There were only four transcription factor genes with FPKM values>10, including *KLF17*, *OLIG3*, *ZFP37* and *ZFP42*, which are critical in the porcine genome activation process. These four genes had very low expression in the SCNT embryos. Among them, *ZFP37* and *ZFP42* belong to the zinc finger proteins and are closely related to the regulation of gene expression, which is influenced by various microRNAs and can be associated with abnormalities and impairment during embryonic development.

At the 8-cell stage, 1398 DEGs were identified between the NT-D and IV-D groups, including 420 upregulated and 978 downregulated genes, with only 669 DEGs that had gene names in Sscrofa11.1 reference genome (Table S14). A total of 18 nuclear transcription factor genes (with FPKM values>10) were screened out, of which *HLTF*, *PCBD1*, *SMAD5*, *SUV39H2*, *ZHX1* and *ZNF37A* were induced, and *ASZ1*, *FOS*, *HES1*, *HEXIM2*, *HMGB1*, *KLF10*, *NCOA7*, *SNAI1*, *TEAD4*, *TGIF1*, *VGLL2* and *ZFP42* were suppressed. The abnormal expression of these genes can likely cause dysregulation of a large number of downstream genes in the cloned 8-cell embryos. For instance, the transcription factor *TEAD4* has been shown to be necessary for trophectoderm lineage specification (Yagi et al., 2007). IPA canonical pathway analysis revealed that the top enriched canonical pathways were the cholesterol biosynthesis-related pathways, which was consistent with KEGG analysis also showing that steroid biosynthesis was the only pathway that achieved a significant level of enrichment.

At the blastocyst stage, there were 237 DEGs between the NT-D and IV-D groups, including 28 upregulated and 209 downregulated genes, with only 73 genes that had gene names in the Sscrofa11.1 reference genome (Table S15). There were only five nuclear transcription factor genes (with FPKM values>10), including *ASZ1*, *EHF*, *FOXA2*, *KLF2* and *ZIC5*. The *KLF2* gene is associated with cell proliferation, apoptosis and cell migration (Bialkowska et al., 2017; Wang et al., 2017a). The other four genes are involved in cell differentiation and may be the upstream regulatory factors which can influence ICM and TE differentiation in porcine embryos.

The canonical pathways associated with DEGs in the four different developmental stages between cloned and *in vitro* fertilized Duroc embryos were clustered. The significantly enriched signaling pathways (*P*-value<0.05 and |z-score|>2) included colorectal cancer metastasis signaling, signaling by Rho family GTPases, leukocyte extravasation signaling, IL-8 signaling and Rac signaling pathway (Fig. S3A). All of these terms refer to cellular signaling pathways associated with cancer development and inflammatory responses. With *P*-value<0.05 as the cutoff value, the top five significant pathways for enrichment were cholesterol biosynthesis III, cholesterol biosynthesis II, cholesterol biosynthesis I, hepatic fibrosis/hepatic stellate cell activation and zymosterol biosynthesis pathway (Fig. S3B). We found that fatty acid synthesis and metabolism in the early cleavage process of cloned embryos, especially after reaching the 8-cell stage, as predicted, inhibited relative to normal embryos, indicating that fatty acid biosynthesis in cloned embryos is subject to serious obstacles. The upstream TFs derived from IPA comparison analysis (*P*-value<0.05) included *SMARTA4*, *GATA6*, *RUNX2*, *KLF4*, *CREB1*, *HNF1A*, *UXT*, *PPARGC1A*, *MYCN*, *CREM* and *SMAD7*. These genes mainly clustered in the 8-cell stage and thus may be important functional genes capable of leading to developmental arrest in SCNT embryos during EGA.

### Comparison of expression levels of histone methylation related genes in porcine cloned and fertilized embryos

**Fig. 8.**
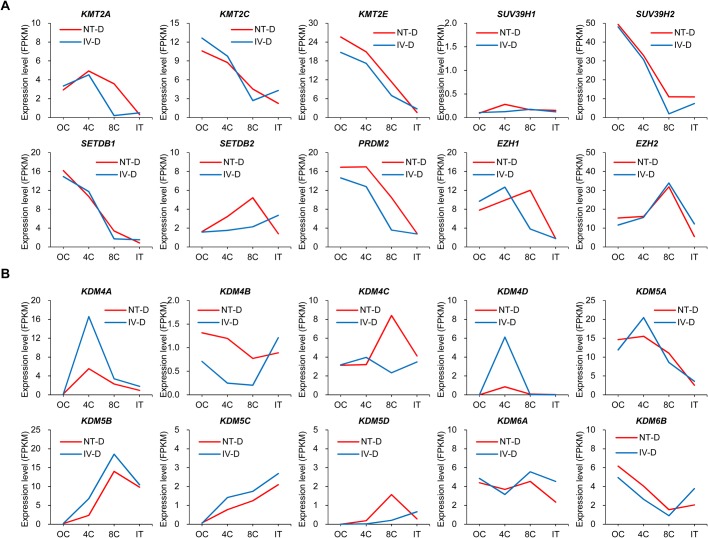
**Expression patterns of histone lysine methylation related genes.** (A) Transcription levels of tri-methyltransferases of H3K4, H3K9 and H3K27 during cloned and *in vivo* fertilized pre-implantation embryos of the Duroc breed. (B) Transcription levels of specific demethylases of H3K4, H3K9 and H3K27 during cloned and *in vivo* fertilized pre-implantation embryos of the Duroc breed.

Histone methylation modifications play an important role in regulation of gene expression and chromatin function. Several studies have reported that dysregulation of tri-methylation modifications of histones H3K9, H3K4 and H3K27 prevent efficient reprogramming of mouse cloned embryos (Hörmanseder et al., 2017; Matoba et al., 2014, 2018). Histone lysine methyltransferases (KMTs) and histone lysine demethylases (KDMs) tightly regulate methylation of histone lysine residues to maintain cell fate and genomic stability and play complex roles in transcription, replication and cell division (Black et al., 2012; Dimitrova et al., 2015; Marmorstein and Trievel, 2009). Given the importance of KMTs and KDMs for regulation of embryo development and cell reprogramming, we used our transcriptome sequencing data to investigate differences in expression of KMTs and KDMs between cloned and fertilized porcine embryos (Table S16). We focused on the expression of KMT and KDM genes related to H3K4me3, H3K9me3 and H3K27me3 with available accession numbers in the Sscrofa11.1 reference genome. Specifically, *KMT2A*, *KMT2C* and *KMT2E* are H3K4 tri-methyltransferases; *SUV39H1*, *SUV39H2*, *SETDB1*, *SETDB2* and *PRDM2* are H3K9 tri-methyltransferases; and *EZH1* and *EZH2* are related to H3K27me3. We compared the FPKM values for the above ten genes in all the samples in the 27-sample dataset (Fig. 8A). The expression levels of *KMT2A* and *SUV39H2* were significantly higher in the cloned 8-cell embryos compared to the fertilized 8-cell embryos, while the other genes were not significantly different between these two types of embryos during the same cleavage stage. H3K4me3 and H3K9me3-related KMTs were highly expressed in the oocytes and 4-cell embryos, and showed a rapid decline in expression in the 8-cell embryos, indicating that the two types of histone methylation modifications are established early and may require extensive demethylation during porcine embryo activation. The expression of *EZH2* gene was significantly increased in the cloned and fertilized 8-cell embryos, indicating that the 8-cell stage may be an important period for establishing H3K27me3 modification. *SUV39H1* is an important H3K9me3 modifying enzyme in mouse embryos (Matoba et al., 2014), but it was not expressed in mature porcine oocytes or in early embryos as estimated from our sequencing data. The genes in the KDM5 family, KDM5A–5D (JARID1A–1D), specifically remove H3K4 di- and tri-methylation. The KDM4 family includes four homologous demethylases, KDM4A–4D (JMJD2A–2D, respectively) (Zhang et al., 2012). KDM4 proteins are able to remove H3K9 di- and tri-methylation, while KDM6A and KDM6B are H3K27-specific demethylases, which can remove H3K27 di- and tri-methylation. Comparing the FPKM values of KDM genes for all samples in the 27-sample dataset (Fig. 8B), the expression of *KDM4A*, *KDM4D* and *KDM5B* was significantly lower in the cloned 4-cell embryos compared to the fertilized 4-cell embryos. The expression of other genes was comparable between these two types of embryos at the same cleavage period. Both in the cloned and fertilized embryos, *KDM4A*, *KDM4D* and *KDM5A* were expressed at the highest level during the 4-cell stage, and *KDM5B* reached the highest expression level during the 8-cell stage, indicating that the demethylation of H3K4 and H3K9 in porcine early embryos occurs mainly in the 4- and 8-cell stages, and the demethylation of H3K9 occurs sooner. Previous studies have shown that *KDM4B* can play an important role in mouse somatic cell reprogramming (Liu et al., 2016; Wei et al., 2017), but it was lowly expressed during porcine early embryonic development according to our data.

## DISCUSSION

Multi-omics sequencing of various species has developed rapidly, and several recent studies have reported the gene expression dynamics of human and mouse pre-implantation embryos by RNA-Seq, which has important implications for the study of embryonic development mechanisms (Petropoulos et al., 2016; Stirparo et al., 2018; Xue et al., 2013; Yan et al., 2013). However, in the study of porcine embryo development, there are very few reports highlighting the whole genome gene expression regulatory mechanism of the pre-implantation of cloned embryos or normal embryos. Here, we utilized RNA-Seq to systematically characterize the transcriptome profiles of porcine matured oocytes developing into the blastocyst stage. The developmental efficiency of cloned embryos may differ from that of *in vitro* culture conditions, but to avoid any additional stress on cloned embryos due to surgical transplantation and embryo flushing from the porcine uterus, we cultured the cloned embryos *in vitro* and rigorously selected embryos with better morphology, no fragmentation and homogeneous blastomeres for the construction of sequencing libraries (Cagnone and Sirard, 2016; Østrup et al., 2013). Quality control after cDNA amplification during the construction of all the sequencing libraries showed normal peaks and no RNA degradation. Consequently, our cloned embryo samples were suitable for studying the typical characteristics and defects in gene expression in the cloned embryos at each specific cleavage period. In this study, *in vivo* fertilized embryos were used as a control group. Utilizing fertilized embryos offers advantages, as the development efficiency of *in vivo* derived porcine embryos is higher compared to *in vitro* fertilized embryos, which are commonly used as controls in multiple publications (Bauer et al., 2010; Lee et al., 2018). In pigs, the efficiency of *in vitro* fertilization is very low because of several problems, such as incomplete maturation of oocytes after *in vitro* maturation, a high incidence of polyspermy and poor quality of blastocysts (Grupen, 2014). Additionally, although it is technically feasible to perform RNA-Seq on a single embryo or even a single cell, for this experiment, we decided to prepare each sample with about ten oocytes or embryos. Gene expression patterns between the individual embryos may exhibit individual differences, and some embryos may have cytogenetic abnormalities, such as gene mutations, which may directly affect their gene expression profiles. Transcriptome analysis of a single embryo or of a single blastomere may reflect cellular abnormalities within that particular embryo. Without many biological duplicates, using multiple mixed embryos is more suitable for the analysis of transcriptome characteristics at a particular developmental stage (Graf et al., 2014). Since the sample preparation had to be completed in a very short time, we did not conduct gender identification for IV-D embryos. All male and female embryos of the same cleavage period collected from a pregnant sow were mixed into a single sample for sequencing. However, it is important to note that the NT-LW and NT-D embryos collected for RNA-Seq were all male. Gender differences between the three groups of embryos may also result in differences in gene expression, especially for genes located on the sex chromosomes. Based on the analysis of our transcriptomic data of single 4-cell embryos (unpublished data), the sex of *in vivo* fertilized 4-cell embryos mainly influenced gene expression levels on the sex chromosomes, but other autosomal genes were not affected. Therefore, we only considered the influence of gender on sex chromosomes. The results described above indicate that the RNA integrity of our embryonic samples was high, resulting in good quality in the sequencing libraries. The mapping rate of all sequenced clean reads to the reference genome was more than 90%, and the average number of genes detected more than 10,000 (with FPKM value>1). These results are comparable with other single-cell RNA-Seq methods reported in previous studies (Biase et al., 2014; Wang et al., 2017b), indicating that the transcriptome sequencing method used in this experiment is likely valid, the reliability and accuracy of the transcriptome data are high and this analysis has the potential to provide meaningful reference data for future research.

After the donor cells were transferred to the recipient oocyte, the cloned embryos underwent nuclear silencing of gene expression, epigenetic memory erasure in somatic cells, embryonic genome activation and reestablishment of new epigenetic modifications (Saitou et al., 2012; Xu and Xie, 2018). Donor cells determine the correctness and completeness of the nuclear reprogramming process, which is one of the most critical factors affecting cloning efficiency. Many previous studies have shown that donor cell type, differentiation, passage number and epigenetic modification state can all influence cloning efficiency (Bonk et al., 2007; Hirasawa et al., 2013; Zhai et al., 2018). Using several years of statistical analyses of pig cloning experimental data, our research team discovered that the cloning efficiency of Laiwu pigs was significantly higher compared to that of Duroc pigs (Li et al., 2013c). Laiwu pigs are one of the most prolific pig breeds in China. Duroc sires imported from the United States are utilized most frequently as a terminal/paternal sire in the three-breed terminal cross-breeding program. Duroc boars are the predominant terminal sires worldwide, and they provide 100% heterosis when mated to Yorkshire×Landrace F1 females. According to the results of the preliminary experiments, the blastocyst development efficiency of the Laiwu and Duroc cloned 1-cell embryos collected in our sequencing experiment reached 30% and 20%, respectively, indicating that a significant difference exists between the cloning efficiency of the two donor cell lines. In this study, we compared the differences in gene expression patterns of NT-LW and NT-D embryos. We sought to identify the intrinsic molecular mechanisms that underlie differences in cloning efficiency between these two different breeds of pigs. Our analysis indicated that the number of genes that failed to be appropriately activated in the 4-cell NT-LW embryos was significantly lower compared to the NT-D embryos. Another important feature of the NT-LW embryos is that the pattern of pluripotent gene expression changes from the cloned 1-cell embryo to the blastocyst stage is more similar to a normally fertilized embryo. In particular, the variation tendency of *MYC*, *NANOG*, *KLF4*, *BMP4* and *POU5F1* in the early development of the NT-LW embryos and normal embryos was consistent while the dysregulation of pluripotent genes in the NT-D embryos was more variable. These pluripotent genes are key candidates that can drive the development of early embryos, which can likely explain the higher developmental potential of NT-LW embryos.

In the present study, we obtained the dynamic transcriptional profiles of porcine cloned and *in vivo* fertilized embryos. Our data provide comprehensive insight into the aberrant gene expression patterns in porcine cloned embryos produced via SCNT. The profiling of DEGs, identified pathways and key TFs will facilitate in-depth understanding the molecular mechanisms underlying the failure of SCNT. The dysregulated critical genes observed in cloned embryos could serve as potential candidate genes for the embryonic competence gene markers selection and verification. We identified many previously unknown genes abnormally expressed in cloned porcine embryos at seven cleavage stages. Dozens of TFs were found to be abnormally expressed in cloned porcine embryos at specific stages, of which several TFs (*MYC*, *TP53*, *TET1*, *MYCN*, *SUV39H2*, *KDM4A*, *KDM4D*, *KDM5B*) have been reported to play essential roles in mouse and human early cloned embryo development, but are not well understood in pigs (Liu et al., 2016; Matoba et al., 2014; Matoba and Zhang, 2018). We have found the top significant pathways associated with the DEGs in cloned and normal embryos to include oxidative phosphorylation, mitochondrial dysfunction, protein ubiquitination and cholesterol biosynthesis. In addition, we focused on genes related to histone lysine methylation, which is a hot topic in the study of improving mouse SCNT efficiency in recent years. For this reason, we provide evidence that H3K4 and H3K9 methylation contribute to resistance to transcriptional reprogramming in cloned porcine embryos.

In summary, for the first time, we reported the whole genome transcriptomes of the three sources of porcine pre-implantation embryos, including Laiwu and Duroc cloned embryos and *in vivo* fertilized Duroc embryos. Our data not only provided a valuable resource for dissection of gene regulatory mechanisms underlying the development of porcine embryos, but also identified novel molecular defects and transcriptome changes during SCNT reprogramming. Further work based on these results might significantly increase the efficiency of pig SCNT technology.

## MATERIALS AND METHODS

### Animal ethics statement

This study was carried out in strict accordance with ‘The Instructive Notions with Respect to Caring for Laboratory Animals’ issued by the Ministry of Science and Technology of China. The animal experimental protocol was approved by the Institutional Animal Care and Use Committee of South China Agricultural University. All efforts were made to minimize animal suffering.

### Donor cell culture and somatic cell nuclear transfer

Nuclear donor cells of the NT-LW and NT-D embryos were isolated from ear fibroblasts of a 1-year-old Laiwu boar and a 1-year-old Duroc boar, respectively. The Duroc boar was the male parent of all the RNA sequencing fertilized embryos. The primary cells were frozen in liquid nitrogen. Prior to SCNT, donor cells were thawed and cultured in Dulbecco's modified eagle medium (DMEM) supplemented with 10% fetal bovine serum at 37°C in a humidified atmosphere of 5% CO_2_ and 95% air. Cells at three to five passages were used for this study.

Porcine ovaries were collected from a local slaughterhouse in Tianhe District, Guangzhou. Cumulus-oocyte complexes (COCs) were picked out from the follicular fluid and cultured *in vitro* for 42–44 h. Matured COCs were aspirated into a 1.5 ml tube and freed from cumulus cells by repeated pipetting in 0.1% hyaluronidase. Matured oocytes with a first polar body were selected as nuclear receptors.

The SCNT experiments were performed according to our previous study (Li et al., 2013b). After the micromanipulation of removing oocyte nucleus, we performed staining with Hoechst 33342 fluorescent dye (Thermo Fisher Scientific) and visualized the cells with subsequent short UV exposure to confirm successful enucleation. Oocytes which still had nuclei were discarded (Iuso et al., 2013). A single fibroblast cell was microinjected into the perivitelline space of the oocytes. The oocyte-donor cell complexes were cultured in the PZM3 medium at 38.5°C for 30 min and then activated to fuse by two successive DC pulses of 1.2 kV/cm for 30 μs using an electro-fusion instrument (model: CF-150/B, BLS Company, Hungary). The reconstructed embryos were cultured in a low-oxygen humidified atmosphere containing 5% CO_2_, 5% O_2_ and 90% N_2_ at 38.5°C.

### Collection of porcine oocytes and embryos for RNA sequencing

*In vivo* matured oocytes were collected from 2-year-old Duroc sows in estrus. *In vivo* fertilized embryos at different stages were collected from pregnant Duroc sows after artificial insemination. The male parent of all IV-D embryos was the same Duroc boar, resulting in all IV-D embryos being classified as paternal half-siblings. The embryos were flushed from the uteri after post-mortem hysterectomy. Briefly, the front uterine horns were first clamped with hemostatic forceps, Dulbecco's phosphate-buffered saline (DPBS) containing 1% PVA was injected from the tubal fimbria into the uterus, collected at the cervix with a plastic catheter, and then finally embryos were collected under a stereoscopic microscope. The embryonic selection criteria were based on the previous studies (Baczkowski et al., 2004; Yan et al., 2013). The selection of SCNT embryos for RNA-Seq followed similar screening criteria as for normal embryos. About five to ten cloned embryos at the same cleavage stage with blastomeres of similar size and no intracytoplasmic fragments were mixed to form a SCNT embryo sample. All embryos selected for this study had good morphology. The zona pellucida of oocytes and embryos was removed by acidic Tyrode's solution (Sigma-Aldrich, T1788), and then washed twice with DPBS containing 0.1% bovine serum albumin before placing in lysis buffer. Since the porcine ICM and TE are difficult to separate while maintaining cell viability, a hand-held microsurgical knife was used to cut a blastocyst into pure TE and a part of the embryonic cell containing ICM and TE under a stereoscopic microscope (Cao et al., 2014). By comparing the transcriptome data of these two parts of blastocysts, approximate gene expression profiles of the ICM and TE cells can be obtained.

### RNA library construction and sequencing

Cells, oocytes and embryos were lysed in 6 μl of lysis buffer with RNase inhibitor. cDNA was generated and amplified with the SMART-Seq^®^ v4 Ultra Low Input RNA Kit for Sequencing (Clontech, 634892). Sequencing libraries were generated using NEBNext Ultra RNA Library Prep Kit for Illumina following manufacturer's recommendations and index codes were added to attribute sequences to each sample. Paired-end 150 bp sequencing was performed on an Illumina Hiseq platform at Novogene Corporation in Beijing.

### Reads mapping and gene expression analysis

Reference genome (Sscrofa11.1) and gene annotation files were downloaded from Ensembl public FTP site (http://asia.ensembl.org/info/data/ftp/index.html). Paired-end clean reads were aligned to the porcine reference genome using Hisat2 v2.0.4. HTSeq v0.9.1 was used to count the reads numbers mapped to each gene. Gene expression level was measured as FPKM to eliminate the effects of sequencing depth and transcript length. We identified all of the genes with a FPKM value ≥1 as the expressed genes.

### Differential expression analysis

For the 25 sequencing samples without biological replicates, prior to differential gene expression analysis, for each sequenced library, the read counts were adjusted by edgeR program package through one scaling normalized factor. Differential expression analysis was performed using the DEGSeq R package 1.20.0. The *P*-values were adjusted using the Benjamini-Hochberg procedure. Corrected *P*-value of 0.005 and log_2_(fold-change) of 1 were set as the threshold for significantly differential expression.

For the 27 sequencing samples with three biological replicates per group, DEGs were identified using DESeq R package 1.18.0. DESeq provides statistical routines for determining differential expression in digital gene expression data using a model based on the negative binomial distribution (Anders and Huber, 2010). The resulting *P*-values were adjusted using the Benjamini-Hochberg procedure for controlling the false discovery rate. Genes with an adjusted *P*-value<0.05 were considered to be differentially expressed.

Principal component analysis for RNA-Seq samples was implemented using R function prcomp, and only genes with averaged FPKM value ≥1 were used for the analysis. FPKM values for the differentially expressed genes were log10-transformed and reordered according to hierarchical clustering using the R packages. The sample correlation matrix was investigated using the squared Pearson correlation coefficient (R^2^) between each possible pair of samples.

### Functional analysis of DEGs

GO enrichment analysis of DEGs was performed using the goseq v1.24.0 in R. GO terms with corrected *P*-values<0.05 were considered statistically significant. We also used KOBAS 3.0 software to test the statistical enrichment of DEGs in the KEGG pathways.

We used Ingenuity Pathway Analysis (IPA) software to identify significantly enriched pathways for DEGs between different points in time and different sources of embryos. Only DEGs with gene symbols were identified by IPA datasets for performing ‘core analysis’. IPA was used to identify top canonical pathways, upstream regulators, mechanistic networks, and gene interaction networks. ‘Comparison analysis’ is a powerful functional clustering tool for analyzing multiple groups of embryos. Calculating the z-score can help infer the activation states (‘activated’ or ‘inhibited’) of implicated biological processes. Significance was determined using a Fisher's exact *P*-value that was adjusted for multiple testing correction using the Benjamini-Hochberg method. A detailed description of the method for using IPA can be found at www.ingenuity.com.

## Supplementary Material

Supplementary information
