# Supplementary material for: Characterization and comparative analyses of transcriptomes of cloned and in vivo fertilized porcine pre-implantation embryos
Source: Biol Open. 2019 Apr 5;8(4):bio039917. doi: 10.1242/bio.039917 (PMC6504007; doi:10.1242/bio.039917)
Supplement: Supplementary information [file biolopen-8-039917-s1.pdf]

Fig. S1. Principal component analysis of the transcriptome of Duroc donor cells and embryos in the 27-sample dataset. Samples at the same stage of development cluster closer than they do to any other sample. In addition, the variation among 4-cell embryos was more pronounced than at the other embryo stages.

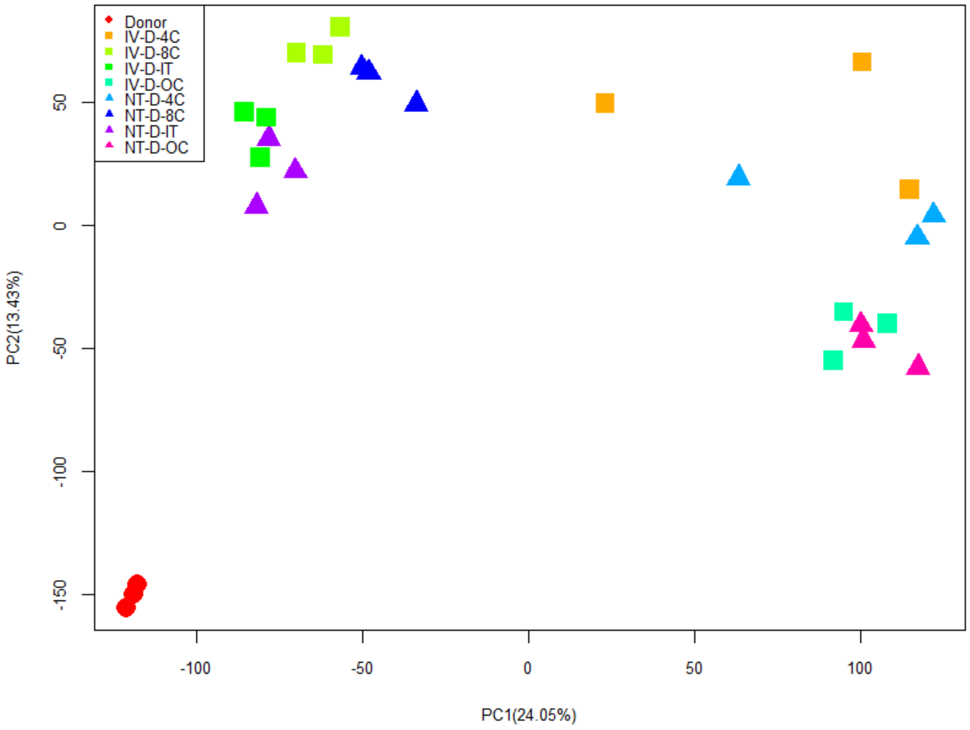

Fig. S2. Heatmaps from results of IPA comparison analysis of DEG core analysis in each two successive stage of three groups of porcine pre-implantation embryos. (A) Over-representation of IPA canonical pathway annotation terms for DEGs between each two successive stages by activity analysis z-scores. Criteria for statistical significance were *P*-value < 0.001 and |z-score| > 2. (B) Over-representation of IPA canonical pathway annotation terms by *P*-values where the criterion for statistical significance was *P*-value < 0.001. (C) Upstream transcription factors identified for the

Biology Open • Supplementary information

sequential development of the successive cleavage stages of porcine embryos. Criteria for statistical significance were P-value < 0.001 and |z-score| > 2.

A

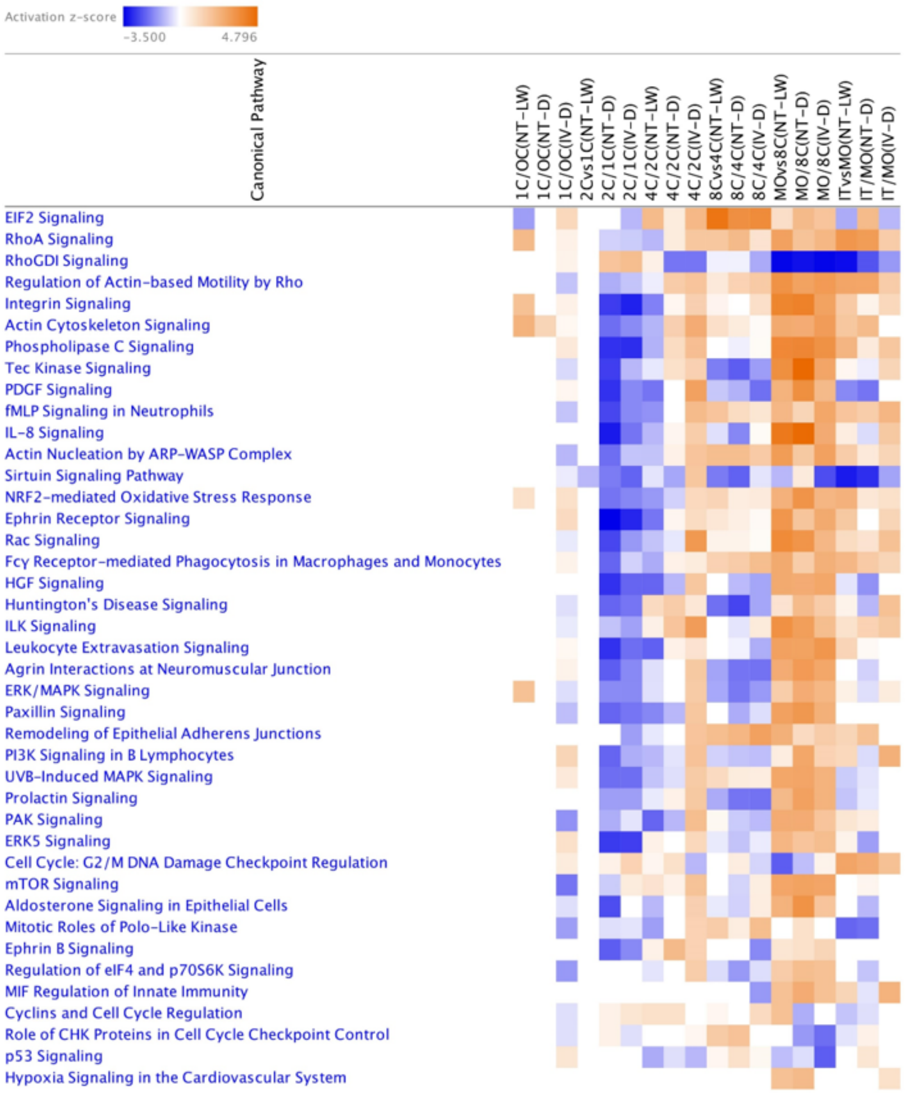

Biology Open • Supplementary information

B

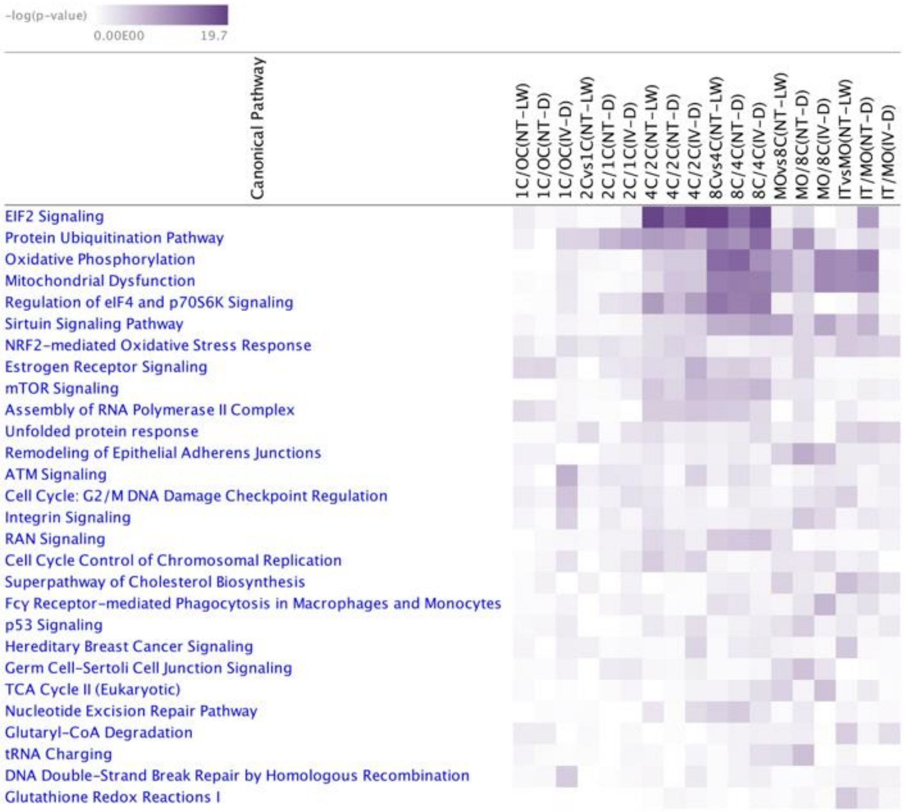

Biology Open • Supplementary information

C

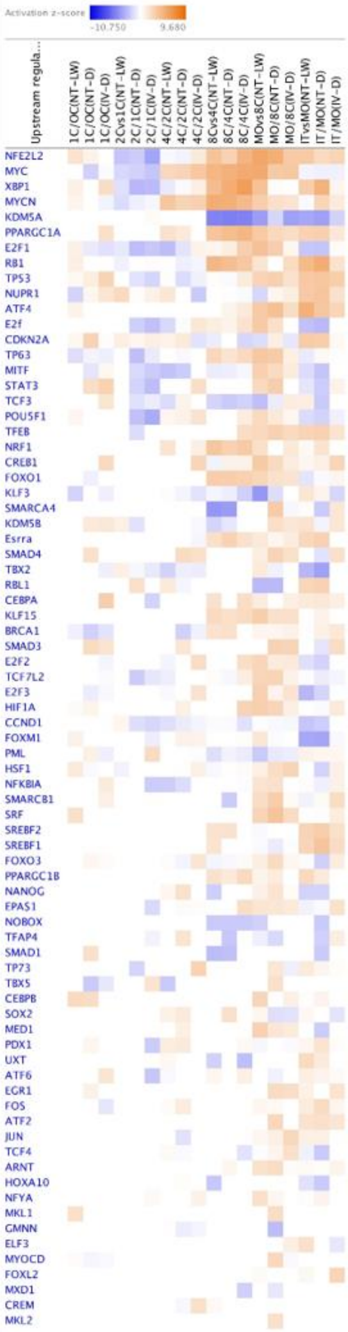

Biology Open • Supplementary information

Fig. S3. Heatmaps from IPA Comparison Analysis results of DEGs core analysis between the cloned and *in vivo* fertilized embryos from the 27-sample dataset. (A) Over-representation of IPA canonical pathway annotation terms for DEGs between the cloned and *in vivo* fertilized embryos at four stages. Criteria for statistical significance were P-value < 0.05 and |z-score| > 2. (B) Over-representation of IPA canonical pathway annotation terms for DEGs between the cloned and *in vivo* fertilized embryos where the criterion for significance was P-value < 0.05.

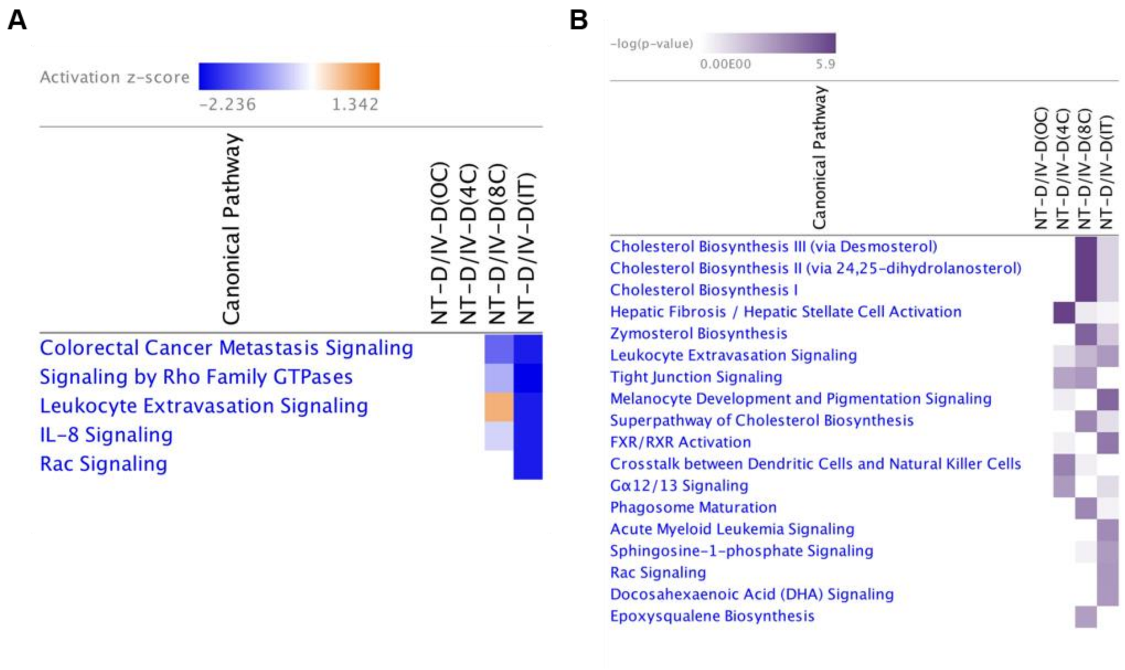

Biology Open • Supplementary information

Supplementary Tables

Table S1: Primary record of samples in the 25-sample dataset.

Click here to Download Table S1

Table S2: Primary record of samples in the 27-sample dataset.

Click here to Download Table S2

Table S3: The sequencing data quality of the 25-sample dataset.

Click here to Download Table S3

Table S4: The sequencing data quality of the 27-sample dataset.

Click here to Download Table S4

Table S5: Summary of filtered reads in the 25-sample dataset aligned to the reference genome.

Click here to Download Table S5

Table S6: Summary of filtered reads in the 27-sample dataset aligned to the reference genome.

Click here to Download Table S6

Table S7: Total number of genes expressed in each sample of the 25-sample dataset.

Click here to Download Table S7

Table S8: Total number of genes expressed in each sample of the 27-sample dataset.

Click here to Download Table S8

Table S9: Linear correlation coefficients (R^2) between samples in the 27-sample dataset.

Click here to Download Table S9

Table S10: Stage-specific genes in each developmental stage of NT-LW, NT-D and IV-D embryos.

Click here to Download Table S10

Table S11: First-expressed genes in each developmental stage of NT-LW, NT-D and IV-D embryos.

Click here to Download Table S11

Table S12: Differentially expressed genes between *in vitro* and *in vivo* mature oocytes.

Click here to Download Table S12

Table S13: Differentially expressed genes between of 4-cell NT-D embryos and 4-cell IV-D embryos.

Click here to Download Table S13

Table S14: Differentially expressed genes between 8-cell NT-D embryos and 8-cell IV-D embryos.

Click here to Download Table S14

Table S15: Differentially expressed genes between cloned blastocysts and *in vivo* derived blastocysts.

Click here to Download Table S15

Table S16: Expression levels of histone lysine methyltransferases and histone lysine demethylases in four developmental stages of NT-D and IV-D embryos.

Click here to Download Table S16

Biology Open • Supplementary information
